# Crystal structures of *N* ^2^,*N* ^3^,*N* ^5^,*N* ^6^-tetra­kis­(pyridin-2-ylmeth­yl)pyrazine-2,3,5,6-tetra­carboxamide and *N* ^2^,*N* ^3^,*N* ^5^,*N* ^6^-tetra­kis­(pyridin-4-ylmeth­yl)pyrazine-2,3,5,6-tetra­carboxamide

**DOI:** 10.1107/S205698901700127X

**Published:** 2017-01-31

**Authors:** Dilovan S. Cati, Helen Stoeckli-Evans

**Affiliations:** a Debiopharm International S.A., Chemin Messidor 5-7, CP 5911, CH-1002 Lausanne, Switzerland; b Insitute of Physics, University of Neuchâtel, rue Emile-Argand 11, CH-2000 Neuchâtel, Switzerland

**Keywords:** crystal structure, pyrazine, pyridine, tetra­carboxamide, hydrogen bonding

## Abstract

The title compounds, C_32_H_28_N_10_O_4_· unknown solvent, (I), and C_32_H_28_N_10_O_4_, (II), are pyrazine-2,3,5,6-tetra­carboxamide derivatives. In (I), the substituents are (pyridin-2-ylmeth­yl)carboxamide, while in (II), the substituents are (pyridin-4-ylmeth­yl)carboxamide. Both compounds crystallize in the monoclinic space group *P*2_1_/*n*, with *Z*′ = 1 for (I), and *Z*′ = 0.5 for (II). The whole mol­ecule of (II) is generated by inversion symmetry, the pyrazine ring being situated about a center of inversion. In (I), the four pyridine rings are inclined to the pyrazine ring by 83.9 (2), 82.16 (18), 82.73 (19) and 17.65 (19)°. This last dihedral angle involves a pyridine ring that is linked to the adjacent carboxamide O atom by an intra­molecular C—H⋯O hydrogen bond. In compound (II), the unique pyridine rings are inclined to the pyrazine ring by 33.3 (3) and 81.71 (10)°. There are two symmetrical intra­molecular C—H⋯O hydrogen bonds present in (II). In the crystal of (I), mol­ecules are linked by N—H⋯O and N—H⋯N hydrogen bonds, forming layers parallel to (10-1). The layers are linked by C—H⋯O and C—H⋯N hydrogen bonds, forming a three-dimensional framework. In the crystal of (II), mol­ecules are linked by N—H⋯N hydrogen bonds, forming chains propagating along the [010] direction. The chains are linked by a weaker N—H⋯N hydrogen bond, forming layers parallel to the (101) plane, which are in turn linked by C—H⋯O hydrogen bonds, forming a three-dimensional structure. In the crystal of compound (I), a region of disordered electron density was treated with the SQUEEZE routine in *PLATON* [Spek (2015 ▸). *Acta Cryst*. C**71**, 9–18]. Their contribution was not taken into account during refinement. In compound (II), one of the pyridine rings is positionally disordered, and the refined occupancy ratio for the disordered C_ar_—C_ar_—N_py_ atoms is 0.58 (3):0.42 (3).

## Abstract 2

The two title compounds are pyrazine-2,3,5,6-tetra­carboxamide derivatives. In the first crystal, mol­ecules are linked by N—H⋯O and N—H⋯O hydrogen bonds, forming a two-dimensional network structure. In the second crystal, an N—H⋯N hydrogen bond links the mol­ecules into a chain structure, while a further weak N—H⋯N hydrogen bond links the chains, forming a two-dimensional network structure.

## Chemical context   

Tetra­kis-substituted pyrazine ligands for coordination chemistry, excluding tetra­methyl­pyrazine or pyrazine-2,3,5,6-tetra­carbo­nitrile, are almost exclusively limited to tetra­kis­(2′-pyrid­yl)pyrazine (**tppz**) and tetra­kis­(carb­oxy­lic acid)pyrazine (**H4pztc**). **Tppz** was first synthesized by Goodwin & Lions (1959 ▸). The crystal structure of the first coordination compound of **tppz** to be reported was a binucluear copper(II) complex, bis­{di­aqua­[μ^2^-2,3,5,6-tetra­kis­(2-pyrid­yl)pyrazine-*N*,*N*′,*N*′′,*N*′′′,*N*′′′′,*N*′′′′′]copper(II)} tetra­perchlorate dihydrate, with the ligand coordinating in a bis-tridentate manner (Graf *et al.*, 1993 ▸). **H4pztc** is a much older compound, whose synthesis was first reported by Wolf (1887 ▸, 1893 ▸). The first published complex of **H4pztc** is a one-dimensional iron(II) coordination polymer, *catena*-[μ^2^-(2,5-di­carb­oxy­pyrazine-3,6-di­carboxyl­ato-*N*,*O*)-*trans*-di­aqua­diiron(II)] dihydrate (Marioni *et al.*, 1986 ▸), in which the ligand coordinates in a bis-bidentate manner. There are of course a number of complexes in which **H4pztc** coordinates in a bis-tridentate manner (Cambridge Structural Database; Groom *et al.*, 2016 ▸). Recently, the first pyrazine-2,3,5,6-tetra­carboxamide ligand was reported, namely, *N*,*N*′,*N*′′,*N*′′′-tetra­ethyl­pyrazine-2,3,5,6-tetra­carboxamide, together with its binculear palladium(II) acetate complex (Lohrman *et al.*, 2016 ▸), in which the ligand coordinates in a bis-tridentate manner.
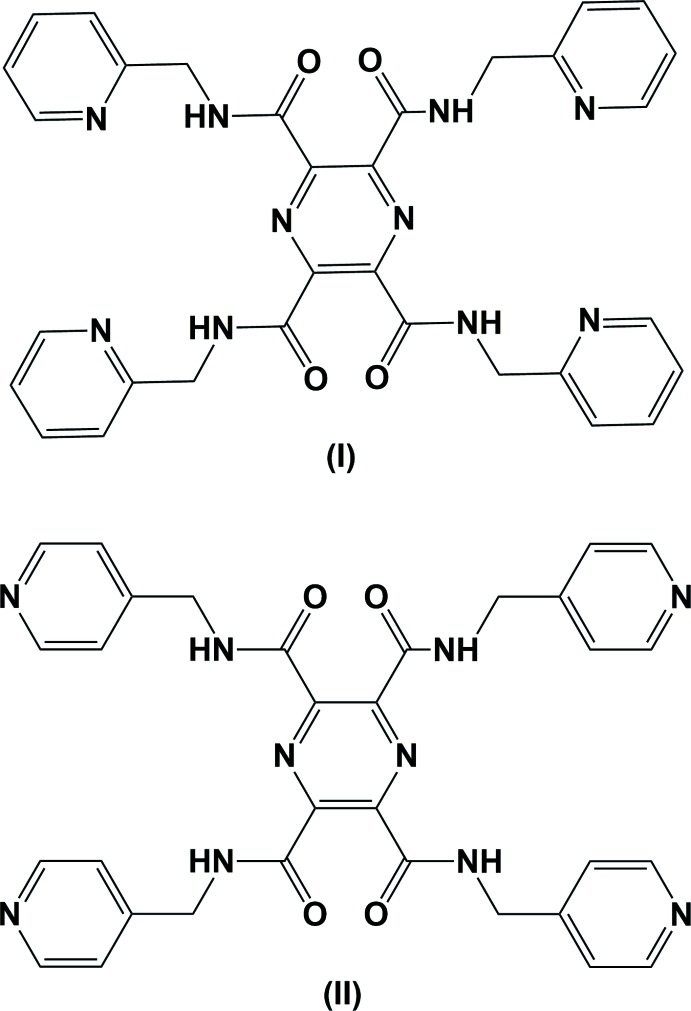


The title compounds are part of a series of mono-, bis- and tetra­kis-substituted carboxamide pyrazine ligands synthesized in order to study their coordination chemistry with first row transition metals and the magnetic exchange properties of the complexes (Cati, 2002 ▸; Cati *et al.*, 2004 ▸). One such ligand is *N*,*N*′-bis­(2-pyridyl­meth­yl)pyrazine-2,3-dicarboxamide, for which two polymorphs have been reported: ortho­rhom­bic (Cati & Stoeckli-Evans, 2004 ▸) and triclinic (Cati *et al.*, 2004 ▸). The reaction of this ligand with copper perchlorate and nickel chloride lead to the formation of [2×2] grid-like structures (Cati *et al.*, 2004 ▸), with multiple encapsulation of the anions. Klingele *et al.* (2007 ▸) have also reported the crystal structures of Cu(BF_4_)_2_ and Ni(BF_4_)_2_ complexes of the same ligand, which also form [2×2] grid-like structures, but this time no encapsulation of the anions was observed. Herein, we report on the synthesis and crystal structures of the title pyrazine-2,3,5,6-tetra­carboxamide derivatives, *N^2^,N^3^,N^5^,N^6^*-tetra­kis(pyridin-2-ylmeth­yl)pyrazine-2,3,5,6-tetra­carboxamide (I) and *N^2^,N^3^,N^5^,N^6^*-tetra­kis­(pyridin-4-ylmeth­yl)pyrazine-2,3,5,6-tetra­carboxamide (II), potential bis-tridentate coordinating ligands.

## Structural commentary   

Both title compounds, (I) and (II), crystallize in the monoclinic space group *P*2_1_/*n*, with *Z*′ = 1 for (I), and *Z*′ = 0.5 for (II). The whole mol­ecule of (II) is generated by inversion symmetry; the pyrazine ring being situated about a center of inversion.

**Figure 1:**
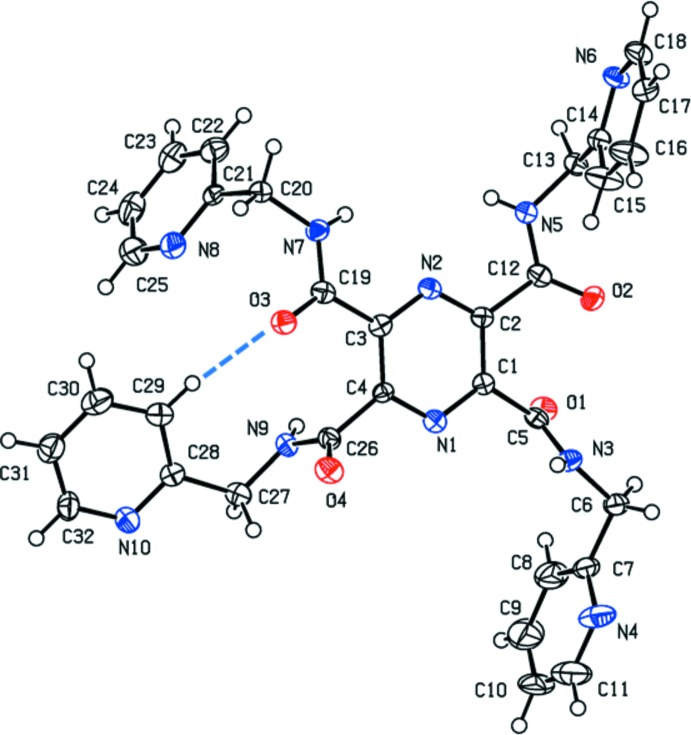
A view of the mol­ecular structure of compound (I), with the atom labelling. Displacement ellipsoids are drawn at the 50% probability level. The intra­molecular C—H⋯O hydrogen bond is shown as a blue dashed line (see Table 1 ▸).

The mol­ecular structure of compound (I), in which the substituents are (pyridin-2-ylmeth­yl)carboxamide, is illustrated in Fig. 1 ▸. Pyridine rings N4/C7–C11, N6/C14–C18 and N8/C21–C25 are inclined to the pyrazine ring by 83.9 (2), 82.16 (18) and 82.73 (19)°, respectively. Pyridine ring N10/C28–C32 is inclined to the pyrazine ring by only 17.65 (19)°, and it is involved in an intra­molecular C29—H20⋯O3 hydrogen bond (Fig. 1 ▸, Table 1 ▸). Adjacent pyridine rings are inclined to one another by 13.7 (2)° for rings N4/C7–C11 and N6/C14–C18, and by 84.5 (2)° for rings N8/C21–C25 and N10/C28–C32.

**Figure 2:**
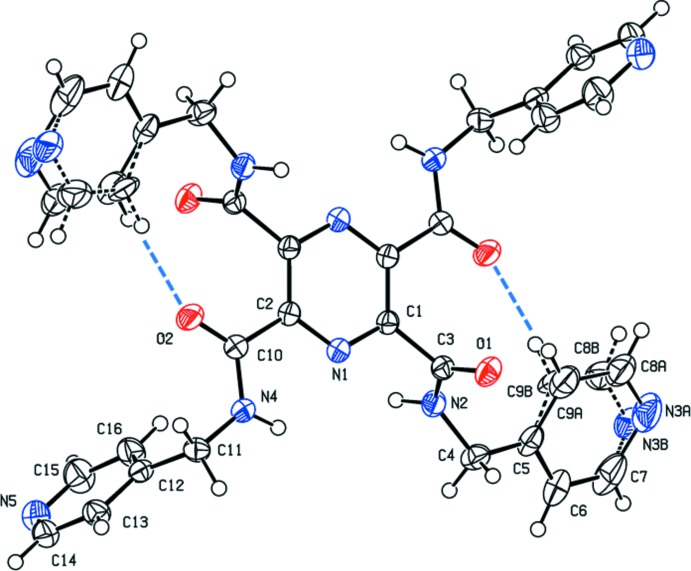
A view of the mol­ecular structure of compound (II), with atom labelling. Displacement ellipsoids are drawn at the 50% probability level. Unlabelled atoms are related to the labelled atoms by the symmetry operation (−*x*, −*y* + 1, −*z* + 2) and the intra­molecular C—H⋯O hydrogen bonds are shown as blue dashed lines (see Table 2 ▸). The minor component of the disordered pyridine ring, involving atom N3, is shown with black dashed lines.

The mol­ecular structure of compound (II), in which the substituents are (pyridin-4-ylmeth­yl)carboxamide, is shown in Fig. 2 ▸. Here, the unique pyridine rings N3*A*/C5–C7/C8*A*/C9*A* [*A* indicates the major component of the disordered atoms] and N5/C12–C16 are inclined to the pyrazine ring by 33.3 (3) and 81.71 (10)°, respectively, and by 68.4 (3)° to one another. In (II) there are also intra­molecular C—H⋯O hydrogen bonds present, as shown in Fig. 2 ▸ (see also Table 2 ▸).

There are no intra­molecular N—H⋯O hydrogen bonds present in either structure and the shortest O⋯O distances, involving adjacent carboxamide groups, are O1⋯O2 = 3.039 (3) Å in (I), and O1⋯O2(−*x*, −*y* + 1, −*z* + 2) = 3.088 (2) Å in (II). In (I), the amide groups in positions 2- and 6- (N3—C5=O1 and N9—C26=O4) are inclined to the pyrazine ring by 67.1 (4) and 83.7 (4)°, respectively, while those in positions 3- and 5- (N5—C12=O2 and N7—C19=O3) are inclined to the pyrazine ring by 14.2 (4) and 21.6 (4)°, respectively.

In (II), the amide group N2—C3=O1, in position 2- (and 5- by symmetry), is inclined to the pyrazine ring by 81.0 (3)°, while the amide group N4—C10=O2, in position 3- (and 6- by symmetry), lies in the plane of the pyrazine ring [dihedral angle = 1.91 (2)°]. Hence, from the various dihedral angles commented on above it can be seen that the conformations of the two mol­ecules are significantly different (*cf*. Fig. 1 ▸ and Fig. 2 ▸).

## Supra­molecular features   

**Figure 3:**
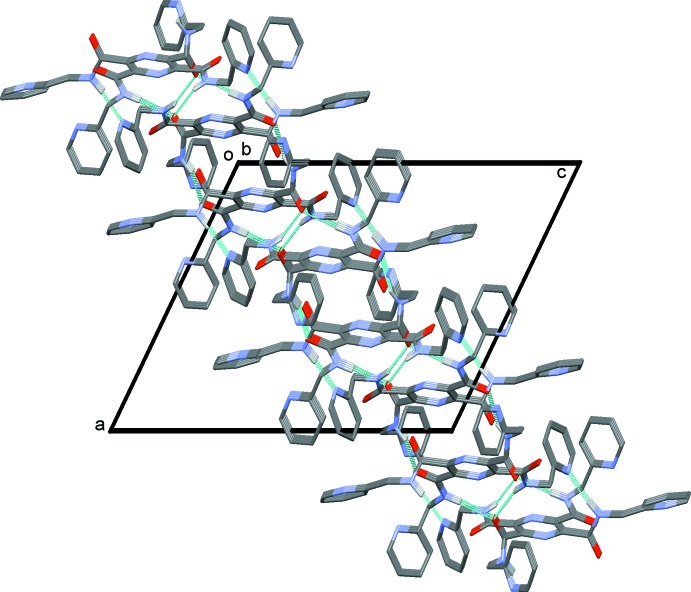
A view along the *b* axis, of the crystal packing of compound (I). The hydrogen bonds are shown as dashed lines (see Table 1 ▸). In this figure, and the following figures, only the H atoms involved in hydrogen bonding have been included.

**Figure 4:**
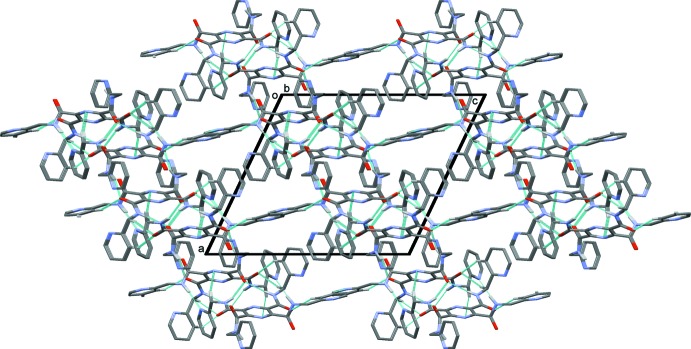
A view along the *b* axis, of the crystal packing of compound (I). The hydrogen bonds are shown as dashed lines (see Table 1 ▸).

In the crystal of (I), mol­ecules are linked by N—H⋯O and N—H⋯N hydrogen bonds, forming layers parallel to (10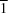); see Fig. 3 ▸ and Table 1 ▸. The layers are linked by C—H⋯O and C—H⋯N hydrogen bonds, forming a three-dimensional framework (Table 1 ▸, Fig. 4 ▸).

**Figure 5:**
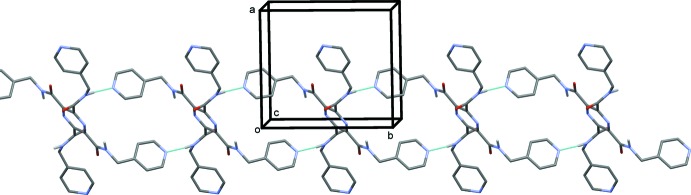
A partial view along the *c* axis, of the crystal packing of compound (II). The hydrogen bonds are shown as dashed lines (see Table 2 ▸).

**Figure 6:**
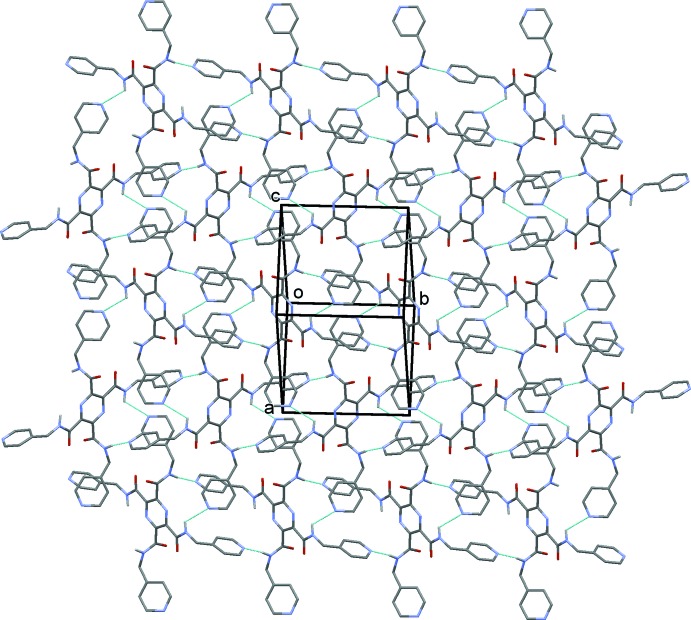
A view normal to plane (101), of the crystal packing of compound (II). The hydrogen bonds are shown as dashed lines (see Table 2 ▸).

**Figure 7:**
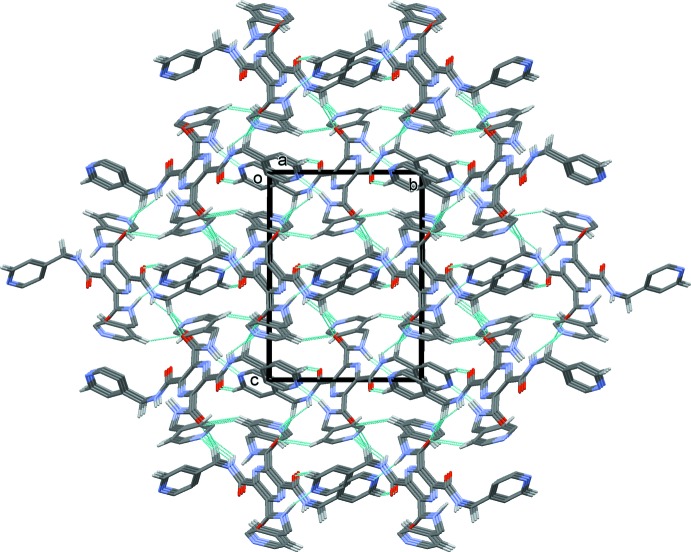
A view along the *a* axis of the crystal packing of compound (II). The hydrogen bonds are shown as dashed lines (see Table 2 ▸).

In the crystal of (II), mol­ecules are linked by N—H⋯N hydrogen bonds (Table 2 ▸), forming chains propagating along [010], as shown in Fig. 5 ▸. The chains are linked by weaker N—H⋯N hydrogen bonds, forming layers (Table 2 ▸, Fig. 6 ▸), parallel to (101). The layers are in turn linked by C—H⋯O hydrogen bonds, forming a three-dimensional framework (Table 2 ▸, Fig. 7 ▸).

## Database survey   

A search of the Cambridge Structural Database (CSD, Version 5.38, first update November 2016; Groom *et al.*, 2016 ▸) for tetra­kis-substituted pyrazines, excluding tetra­methyl­pyrazine or pyrazine-2,3,5,6-tetra­carbo­nitrile, gave over 550 hits. 255 of these structures concern the ligand **tppz**, while 88 concern the ligand **H4pztc**. As noted above, only one example of a pyrazine-2,3,5,6-tetra­carboxamide compound has been reported, *viz. N*,*N*′,*N*′′,*N*′′′-tetra­ethyl­pyrazine-2,3,5,6-tetra­carboxamide (CSD refcode: OSUTIH; Lohrman *et al.*, 2016 ▸). It crystallizes in the triclinic space group *P* 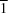, with eleven independent mol­ecules in the asymmetric unit. It is inter­esting to note that the general orientation of the amide groups resembles that observed in compound (I). Those in positions 2- and 6- are inclined to the pyrazine ring by more than *ca* 60 °, while those at positions 3- and 5- lie close to the plane of the pyrazine ring.

## Synthesis and crystallization   

Tetra­methyl pyrazine-2,3,5,6-tetra­carboxyl­ate (**L**) was synthesized by the method of Mager & Berends (1960 ▸).

**Compound (I):** A mixture of **L** (0.16 g, 0.5 mmol) and an excess of 2-(amino­meth­yl)pyridine (0.27 g, 2.5 mmol) in 20 ml of methanol were refluxed for 6 h in a two-necked flask (50 ml). The ligand **H4L8** precipitated as a white solid during the reaction. The suspension was cooled to room temperature and then filtered and washed with 10 ml of cold methanol [yield 90%, m.p. 497 K(decomposition)] . ^1^H NMR (400 MHz, DMSO-*d*_6_): 9.52 (*t*, 1H, *J*_hg_ = 6.1, Hh); 8.53 (*ddd*, 1H, *J*_bc_ = 4.8, *J*_bd_ = 1.8, *J*_be_ = 0.9, Hb); 7.76 (*td*, 1H, *J*_dc_ = 7.7, *J*_db_ = 1.8, Hd); 7.51 (*d*, 1H, *J*_ed_ = 7.8, He); 7.29 (*ddd*, 1H, *J*_cd_ = 7.7, *J*_cb_ = 4.8, *J*_ce_ = 1.0, Hc); 4.64 (*d*, 2H, *J*_gh_ = 6.1, Hg). ^13^C NMR (400 MHz, DMSO-*d*_6_): 164.5, 158.9, 149.7, 146.3, 137.6, 123.2, 122.2, 45.3. IR (KBr pellet, cm^−1^): 3279 (*s*), 3054 (*m*), 1672 (*vs*), 1592 (*vs*), 1571 (*vs*), 1548 (*vs*), 1477 (*s*), 1437 (*vs*), 1354 (*m*), 1290 (*m*), 1247 (*s*), 1179 (*m*), 1157 (*s*), 1099 (*w*), 1049 (*w*), 996 (*m*), 799 (*w*), 754 (*s*), 684 (*m*), 632 (*m*), 608 (*m*), 544 (*w*), 521 (*w*). Analysis for [C_32_H_28_N_10_O_4_]·H_2_O (*M*_r_ = 634.65 g mol^−1^): calculated (%) C: 60.56 H: 4.76 N: 22.07, found (%) C: 60.46 H: 4.58 N: 21.79.

**Compound (II):** This compound was synthesized following the same procedure as used to prepare compound (I). A mixture of **L** (0.5 g, 1.36 mmol) and an excess of 4-(amino­meth­yl)pyridine (1.17 g, 10.8 mmol) were refluxed in 20 ml of methanol for 44 h in a two-necked flask (50 ml). The solution was red when hot and then turned to a brown–yellow colour on cooling to rt. The brown–yellow solid crystallized out, was filtered off and washed with cold aceto­nitrile (m.p. 508 K, yield 90%). ^1^H NMR (400 MHz, DMSO-*d*_6_): 9.50 (*t*, 1H, *J*_hg_ = 6.2, Hh); 8.50 (*dd*, 2H, *J*_ba_ = 4.5, *J*_be_ = 1.6, Hb = Hd); 7.41 (*dd*, 2H, *J*_ab_ = 4.5, *J*_ad_ = *J*_eb_ = 1.6, Ha = He); 4.59 (*d*, 2H, *J*_gh_ = 6.2, Hg). ^13^C NMR (400 MHz, DMSO-*d*_6_): 164.7, 150.4, 148.7, 146.4, 123.1, 42.3. IR (KBr pellet, cm^−1^): 3238 (*s*), 3033 (*m*), 1677 (*vs*), 1604 (*vs*), 1521 (*vs*), 1418 (*vs*), 1364 (*s*), 1317 (*s*), 1239 (*s*), 1174 (*s*), 1151 (*s*), 1069 (*s*), 994 (*s*), 781 (*s*), 616 (*s*), 501 (*w*), 475 (*s*). Analysis for [C_32_H_28_N_10_O_4_]·0.5CH_3_OH (*M*_r_ = 648.68 g mol^−1^): calculated (%) C: 61.10 H: 4.97 N: 21.59, found (%) C: 61.42 H: 4.62 N: 22.27.

Colourless block-like crystals of both compounds were obtained by slow evaporation of methanol solutions of the respective compounds. The elemental analysis for compound (I) required the addition of a water mol­ecule, which possibly explains the region of disordered electron density in the crystal, and half a mol­ecule of methanol for (II), which was not detected in the final difference Fourier map of the crystal used for the X-ray diffraction analysis.

## Refinement   

Crystal data, data collection and structure refinement details are summarized in Table 3 ▸. For both mol­ecules the NH H atoms were located in difference-Fourier maps and freely refined. The C-bound H atoms were included in calculated positions and refined as riding: C—H = 0.95–0.99 Å with *U*_iso_(H) = 1.2*U*_eq_(C). In the crystal of compound (I), a region of disordered electron density was treated with the SQUEEZE routine in *PLATON* (Spek, 2015 ▸). Their contribution (93 electrons for a solvent-accessible volume of 268 Å^3^) was not taken into account during refinement. The crystal of (I) did not diffract significantly beyond 20 ° in θ and hence the *R*_int_ value is high (> 0.2), and only 35% of the data can be considered to be observed [*I* > 2σ(*I*)]. In compound (II), pyridine ring (N3/C5–C9) is positionally disordered (see Fig. 2 ▸), and the refined occupancy ratio for the disordered atoms, N3*A*:N3*B*, C8*A*:C8*B*, C9*A*:C9*B* is 0.58 (3):0.42 (3).

## Supplementary Material

Crystal structure: contains datablock(s) I, II, Global. DOI: 10.1107/S205698901700127X/lh5836sup1.cif

Structure factors: contains datablock(s) I. DOI: 10.1107/S205698901700127X/lh5836Isup2.hkl

Structure factors: contains datablock(s) II. DOI: 10.1107/S205698901700127X/lh5836IIsup3.hkl

Supporting information file. DOI: 10.1107/S205698901700127X/lh5836Isup4.cml

Supporting information file. DOI: 10.1107/S205698901700127X/lh5836IIsup5.cml

CCDC references: 1529572, 1529571

Additional supporting information:  crystallographic information; 3D view; checkCIF report

## Figures and Tables

**Table 1:** Hydrogen-bond geometry (Å, °) for (I)

*D*—H⋯*A*	*D*—H	H⋯*A*	*D*⋯*A*	*D*—H⋯*A*
C29—H29⋯O3	0.95	2.48	3.389 (5)	160
N3—H3*N*⋯O3^i^	0.97 (5)	1.91 (5)	2.829 (4)	158 (4)
N5—H5*N*⋯O1^ii^	0.79 (4)	2.17 (4)	2.932 (4)	162 (3)
N7—H7*N*⋯O1^ii^	0.86 (4)	2.14 (4)	2.967 (4)	161 (4)
N9—H9*N*⋯N6^iii^	0.92 (4)	1.96 (5)	2.864 (4)	169 (5)
C13—H13*A*⋯N1^ii^	0.99	2.62	3.554 (5)	158
C20—H20*A*⋯O2^ii^	0.99	2.54	3.433 (4)	149
C22—H22⋯O2^ii^	0.95	2.57	3.418 (5)	149

Symmetry codes: (i) 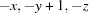; (ii) 
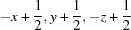
; (iii) 
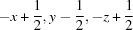
.

**Table 2:** Hydrogen-bond geometry (Å, °) for (II)

*D*—H⋯*A*	*D*—H	H⋯*A*	*D*⋯*A*	*D*—H⋯*A*
C9*A*—H9*A*⋯O2^i^	0.95	2.46	3.316 (15)	150
[C9*B*—H9*B*⋯O2]^i^	0.95	2.43	3.375 (18)	178
N2—H2*N*⋯N5^ii^	0.93 (3)	1.93 (3)	2.845 (3)	167 (2)
N4—H4*N*⋯N3*A* ^iii^	0.90 (3)	2.65 (3)	3.184 (13)	119 (2)
C6—H6⋯O1^iii^	0.95	2.58	3.414 (3)	146
C11—H11*B*⋯O1^iv^	0.99	2.56	3.301 (2)	132
C14—H14⋯O2^v^	0.95	2.58	3.442 (3)	151

Symmetry codes: (i) 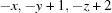; (ii) 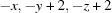; (iii) 
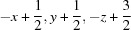
; (iv) 
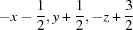
; (v) 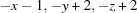.

**Table 3:** Experimental details

	(I)	(II)
Crystal data		
Chemical formula	C_32_H_28_N_10_O_4_	C_32_H_28_N_10_O_4_
*M*_r_	616.64	616.64
Crystal system, space group	Monoclinic, *P*2_1_/*n*	Monoclinic, *P*2_1_/*n*
Temperature (K)	153	153
*a*, *b*, *c* (Å)	16.0754 (19), 11.8602 (10), 18.495 (2)	9.8592 (6), 10.6511 (6), 14.8089 (9)
β (°)	115.503 (13)	102.306 (7)
*V* (Å^3^)	3182.6 (7)	1519.37 (16)
*Z*	4	2
Radiation type	Mo *K*α	Mo *K*α
μ (mm^−1^)	0.09	0.09
Crystal size (mm)	0.40 × 0.20 × 0.20	0.45 × 0.35 × 0.20

Data collection		
Diffractometer	Stoe IPDS 1	Stoe IPDS 1
Absorption correction	Multi-scan (*MULABS* in *PLATON*; Spek, 2009 ▸)	Multi-scan (*MULABS* in *PLATON*; Spek, 2009 ▸)
*T*_min_, *T*_max_	0.865, 1.000	0.666, 1.000
No. of measured, independent and observed [*I* > 2σ(*I*)] reflections	26683, 6150, 2219	11450, 2924, 1815
*R*_int_	0.211	0.090
(sin θ/λ)_max_ (Å^−1^)	0.616	0.614

Refinement		
*R*[*F* ^2^ > 2σ(*F* ^2^)], *wR*(*F* ^2^), *S*	0.054, 0.129, 0.72	0.051, 0.134, 0.89
No. of reflections	6150	2924
No. of parameters	432	244
H-atom treatment	H atoms treated by a mixture of independent and constrained refinement	H atoms treated by a mixture of independent and constrained refinement
Δρ_max_, Δρ_min_ (e Å^−3^)	0.28, −0.30	0.26, −0.24

Computer programs: *EXPOSE*, *CELL* and *INTEGRATE* in *IPDS*-I (Stoe & Cie, 2000 ▸), *SHELXS97* (Sheldrick, 2008 ▸), *Mercury* (Macrae *et al.*, 2008 ▸), *SHELXL2016* (Sheldrick, 2015 ▸), *PLATON* (Spek, 2009 ▸) and *publCIF* (Westrip, 2010 ▸).

## supplementary crystallographic information

### (I) N2,N3,N5,N6-Tetrakis(pyridin-2-ylmethyl)pyrazine-2,3,5,6-tetracarboxamide Crystal data

**Table:**

C_32_H_28_N_10_O_4_	*F*(000) = 1288
*M**_r_* = 616.64	*D*_x_ = 1.287 Mg m^−^^3^
Monoclinic, *P*2_1_/*n*	Mo *K*α radiation, λ = 0.71073 Å
*a* = 16.0754 (19) Å	Cell parameters from 6905 reflections
*b* = 11.8602 (10) Å	θ = 2.1–26.0°
*c* = 18.495 (2) Å	µ = 0.09 mm^−^^1^
β = 115.503 (13)°	*T* = 153 K
*V* = 3182.6 (7) Å^3^	Block, colourless
*Z* = 4	0.40 × 0.20 × 0.20 mm

### (I) N2,N3,N5,N6-Tetrakis(pyridin-2-ylmethyl)pyrazine-2,3,5,6-tetracarboxamide Data collection

**Table:**

Stoe IPDS 1 diffractometer	6150 independent reflections
Radiation source: fine-focus sealed tube	2219 reflections with *I* > 2σ(*I*)
Plane graphite monochromator	*R*_int_ = 0.211
φ rotation scans	θ_max_ = 26.0°, θ_min_ = 2.1°
Absorption correction: multi-scan (MULABS in PLATON; Spek, 2009)	*h* = −19→19
*T*_min_ = 0.865, *T*_max_ = 1.000	*k* = −14→14
26683 measured reflections	*l* = −22→22

### (I) N2,N3,N5,N6-Tetrakis(pyridin-2-ylmethyl)pyrazine-2,3,5,6-tetracarboxamide Refinement

**Table:**

Refinement on *F*^2^	Secondary atom site location: difference Fourier map
Least-squares matrix: full	Hydrogen site location: mixed
*R*[*F*^2^ > 2σ(*F*^2^)] = 0.054	H atoms treated by a mixture of independent and constrained refinement
*wR*(*F*^2^) = 0.129	*w* = 1/[σ^2^(*F*_o_^2^) + (0.0393*P*)^2^] where *P* = (*F*_o_^2^ + 2*F*_c_^2^)/3
*S* = 0.72	(Δ/σ)_max_ < 0.001
6150 reflections	Δρ_max_ = 0.28 e Å^−^^3^
432 parameters	Δρ_min_ = −0.30 e Å^−^^3^
0 restraints	Extinction correction: SHELXL2016 (Sheldrick 2015), Fc^*^=kFc[1+0.001xFc^2^λ^3^/sin(2θ)]^-1/4^
Primary atom site location: structure-invariant direct methods	Extinction coefficient: 0.0015 (3)

### (I) N2,N3,N5,N6-Tetrakis(pyridin-2-ylmethyl)pyrazine-2,3,5,6-tetracarboxamide Special details

**Table:**

Geometry. All esds (except the esd in the dihedral angle between two l.s. planes) are estimated using the full covariance matrix. The cell esds are taken into account individually in the estimation of esds in distances, angles and torsion angles; correlations between esds in cell parameters are only used when they are defined by crystal symmetry. An approximate (isotropic) treatment of cell esds is used for estimating esds involving l.s. planes.

### (I) N2,N3,N5,N6-Tetrakis(pyridin-2-ylmethyl)pyrazine-2,3,5,6-tetracarboxamide Fractional atomic coordinates and isotropic or equivalent isotropic displacement parameters (Å^2^)

**Table:**

	*x*	*y*	*z*	*U*_iso_*/*U*_eq_	
O1	0.17252 (16)	0.2650 (2)	0.25371 (13)	0.0262 (6)	
O2	0.11731 (16)	0.4901 (2)	0.29838 (13)	0.0270 (6)	
O3	0.15003 (16)	0.6495 (2)	−0.05042 (13)	0.0248 (6)	
O4	0.03328 (16)	0.4184 (2)	−0.10516 (13)	0.0296 (7)	
N1	0.10335 (18)	0.3788 (2)	0.07667 (15)	0.0209 (7)	
N2	0.17209 (18)	0.5881 (2)	0.14404 (15)	0.0196 (7)	
N3	0.0179 (2)	0.2690 (3)	0.17069 (17)	0.0238 (7)	
H3N	−0.029 (3)	0.307 (4)	0.125 (3)	0.084 (17)*	
N4	−0.0867 (2)	0.0255 (3)	0.10199 (18)	0.0424 (10)	
N5	0.1965 (2)	0.6453 (3)	0.29048 (18)	0.0235 (8)	
H5N	0.222 (2)	0.680 (3)	0.269 (2)	0.019 (11)*	
N6	0.1542 (2)	0.8767 (3)	0.39734 (17)	0.0260 (8)	
N7	0.2429 (2)	0.7300 (3)	0.07033 (18)	0.0225 (7)	
H7N	0.265 (3)	0.723 (4)	0.121 (2)	0.053 (14)*	
N8	0.3666 (2)	0.7102 (3)	−0.02182 (18)	0.0329 (8)	
N9	0.1864 (2)	0.3770 (3)	−0.04837 (17)	0.0216 (7)	
H9N	0.236 (3)	0.367 (4)	0.000 (3)	0.082 (17)*	
N10	0.2190 (2)	0.3237 (3)	−0.23133 (17)	0.0308 (8)	
C1	0.1173 (2)	0.4044 (3)	0.15150 (18)	0.0184 (8)	
C2	0.1486 (2)	0.5109 (3)	0.18446 (18)	0.0187 (8)	
C3	0.1582 (2)	0.5635 (3)	0.06870 (19)	0.0197 (8)	
C4	0.1232 (2)	0.4582 (3)	0.03481 (19)	0.0175 (8)	
C5	0.1034 (2)	0.3081 (3)	0.19780 (19)	0.0207 (8)	
C6	−0.0042 (3)	0.1650 (3)	0.2026 (2)	0.0279 (9)	
H6A	−0.065750	0.172843	0.202155	0.033*	
H6B	0.041504	0.154238	0.258835	0.033*	
C7	−0.0039 (3)	0.0627 (3)	0.1541 (2)	0.0294 (10)	
C8	0.0768 (3)	0.0094 (4)	0.1648 (3)	0.0503 (12)	
H8	0.134532	0.036800	0.203162	0.060*	
C9	0.0729 (4)	−0.0836 (4)	0.1195 (3)	0.0677 (16)	
H9	0.127695	−0.121059	0.125562	0.081*	
C10	−0.0118 (5)	−0.1218 (4)	0.0652 (3)	0.0646 (16)	
H10	−0.016508	−0.186399	0.033254	0.078*	
C11	−0.0891 (4)	−0.0651 (4)	0.0579 (3)	0.0578 (15)	
H11	−0.147385	−0.091176	0.019642	0.069*	
C12	0.1531 (2)	0.5463 (3)	0.26412 (19)	0.0221 (9)	
C13	0.2021 (2)	0.6979 (3)	0.36311 (19)	0.0245 (9)	
H13A	0.263666	0.732720	0.391842	0.029*	
H13B	0.195560	0.639111	0.398331	0.029*	
C14	0.1290 (2)	0.7871 (3)	0.34741 (19)	0.0229 (9)	
C15	0.0409 (3)	0.7782 (4)	0.2867 (2)	0.0392 (11)	
H15	0.024542	0.715957	0.250896	0.047*	
C16	−0.0228 (3)	0.8602 (4)	0.2787 (2)	0.0408 (12)	
H16	−0.084386	0.853260	0.238703	0.049*	
C17	0.0024 (2)	0.9522 (3)	0.3285 (2)	0.0269 (9)	
H17	−0.040291	1.010774	0.322741	0.032*	
C18	0.0917 (3)	0.9572 (3)	0.3872 (2)	0.0300 (10)	
H18	0.109726	1.020517	0.421982	0.036*	
C19	0.1827 (2)	0.6525 (3)	0.0241 (2)	0.0223 (9)	
C20	0.2835 (2)	0.8163 (3)	0.0381 (2)	0.0240 (9)	
H20A	0.294428	0.886330	0.070075	0.029*	
H20B	0.240326	0.833907	−0.017922	0.029*	
C21	0.3727 (2)	0.7740 (3)	0.04113 (19)	0.0215 (8)	
C22	0.4560 (3)	0.7938 (3)	0.1066 (2)	0.0312 (10)	
H22	0.458295	0.838750	0.149923	0.037*	
C23	0.5354 (3)	0.7478 (4)	0.1084 (2)	0.0377 (11)	
H23	0.593158	0.761289	0.152609	0.045*	
C24	0.5298 (3)	0.6823 (4)	0.0454 (2)	0.0389 (11)	
H24	0.583563	0.649171	0.045208	0.047*	
C25	0.4446 (3)	0.6654 (4)	−0.0177 (2)	0.0391 (11)	
H25	0.441200	0.619099	−0.060761	0.047*	
C26	0.1097 (2)	0.4191 (3)	−0.04760 (19)	0.0223 (8)	
C27	0.1859 (3)	0.3171 (3)	−0.1166 (2)	0.0271 (9)	
H27A	0.123425	0.285802	−0.147570	0.033*	
H27B	0.228937	0.252544	−0.096491	0.033*	
C28	0.2120 (2)	0.3846 (3)	−0.17289 (19)	0.0227 (9)	
C29	0.2268 (3)	0.5004 (3)	−0.1660 (2)	0.0320 (10)	
H29	0.221046	0.541781	−0.124359	0.038*	
C30	0.2501 (3)	0.5542 (4)	−0.2215 (2)	0.0387 (11)	
H30	0.261980	0.632934	−0.217523	0.046*	
C31	0.2560 (3)	0.4926 (4)	−0.2820 (2)	0.0361 (11)	
H31	0.270793	0.527869	−0.321149	0.043*	
C32	0.2399 (3)	0.3788 (4)	−0.2844 (2)	0.0341 (10)	
H32	0.243853	0.336422	−0.326373	0.041*	

### (I) N2,N3,N5,N6-Tetrakis(pyridin-2-ylmethyl)pyrazine-2,3,5,6-tetracarboxamide Atomic displacement parameters (Å^2^)

**Table:**

	*U*^11^	*U*^22^	*U*^33^	*U*^12^	*U*^13^	*U*^23^
O1	0.0257 (14)	0.0249 (16)	0.0236 (13)	0.0019 (12)	0.0064 (11)	0.0010 (11)
O2	0.0316 (15)	0.0256 (16)	0.0252 (13)	−0.0051 (13)	0.0135 (12)	0.0022 (12)
O3	0.0269 (14)	0.0254 (16)	0.0177 (13)	−0.0006 (12)	0.0053 (11)	0.0021 (11)
O4	0.0226 (14)	0.0369 (17)	0.0216 (13)	0.0002 (12)	0.0021 (11)	−0.0022 (11)
N1	0.0173 (15)	0.0232 (19)	0.0199 (15)	0.0024 (14)	0.0059 (12)	0.0000 (13)
N2	0.0152 (15)	0.0207 (18)	0.0209 (15)	0.0015 (13)	0.0059 (12)	−0.0024 (13)
N3	0.0235 (17)	0.0194 (19)	0.0245 (16)	−0.0016 (15)	0.0067 (14)	0.0016 (14)
N4	0.046 (2)	0.029 (2)	0.0347 (19)	−0.0138 (18)	0.0013 (17)	−0.0013 (16)
N5	0.0273 (18)	0.022 (2)	0.0244 (17)	−0.0036 (16)	0.0138 (15)	−0.0035 (15)
N6	0.0277 (17)	0.0223 (19)	0.0279 (16)	0.0003 (15)	0.0119 (14)	−0.0080 (14)
N7	0.0257 (17)	0.0192 (19)	0.0218 (17)	−0.0020 (15)	0.0095 (14)	0.0009 (14)
N8	0.037 (2)	0.029 (2)	0.0394 (19)	−0.0022 (17)	0.0225 (16)	−0.0027 (15)
N9	0.0240 (17)	0.0223 (19)	0.0192 (16)	0.0021 (15)	0.0101 (14)	−0.0003 (13)
N10	0.0328 (19)	0.034 (2)	0.0282 (17)	−0.0031 (16)	0.0155 (15)	−0.0050 (15)
C1	0.0132 (17)	0.022 (2)	0.0170 (17)	0.0016 (16)	0.0038 (14)	0.0009 (15)
C2	0.0160 (18)	0.018 (2)	0.0207 (18)	0.0020 (16)	0.0064 (14)	−0.0011 (16)
C3	0.0160 (18)	0.020 (2)	0.0206 (18)	0.0017 (16)	0.0050 (14)	0.0021 (15)
C4	0.0155 (18)	0.016 (2)	0.0209 (18)	0.0001 (16)	0.0075 (14)	0.0000 (15)
C5	0.024 (2)	0.015 (2)	0.0237 (19)	0.0059 (17)	0.0107 (17)	−0.0020 (15)
C6	0.035 (2)	0.023 (2)	0.0256 (19)	−0.0068 (19)	0.0124 (18)	−0.0025 (16)
C7	0.035 (2)	0.025 (2)	0.026 (2)	−0.011 (2)	0.0107 (17)	−0.0034 (17)
C8	0.046 (3)	0.037 (3)	0.078 (3)	−0.008 (2)	0.037 (2)	−0.018 (3)
C9	0.089 (4)	0.032 (3)	0.110 (4)	−0.007 (3)	0.070 (4)	−0.019 (3)
C10	0.121 (5)	0.025 (3)	0.062 (3)	−0.030 (3)	0.052 (3)	−0.020 (2)
C11	0.083 (4)	0.036 (3)	0.041 (3)	−0.020 (3)	0.015 (3)	−0.011 (2)
C12	0.0189 (18)	0.025 (2)	0.0177 (18)	0.0023 (17)	0.0034 (15)	−0.0007 (16)
C13	0.025 (2)	0.028 (2)	0.0184 (18)	−0.0028 (17)	0.0075 (15)	−0.0034 (15)
C14	0.024 (2)	0.022 (2)	0.0224 (18)	−0.0007 (17)	0.0094 (15)	−0.0014 (16)
C15	0.034 (2)	0.032 (3)	0.037 (2)	0.005 (2)	0.0007 (19)	−0.0133 (19)
C16	0.027 (2)	0.038 (3)	0.039 (2)	0.005 (2)	−0.0025 (18)	−0.014 (2)
C17	0.027 (2)	0.024 (2)	0.0287 (19)	−0.0009 (18)	0.0104 (17)	0.0002 (17)
C18	0.032 (2)	0.026 (2)	0.032 (2)	0.003 (2)	0.0143 (18)	−0.0066 (17)
C19	0.0191 (19)	0.022 (2)	0.024 (2)	0.0024 (17)	0.0075 (16)	−0.0022 (16)
C20	0.029 (2)	0.020 (2)	0.0253 (19)	−0.0029 (17)	0.0137 (17)	−0.0028 (16)
C21	0.027 (2)	0.015 (2)	0.0255 (19)	−0.0026 (17)	0.0144 (16)	0.0014 (16)
C22	0.033 (2)	0.025 (2)	0.033 (2)	−0.0001 (19)	0.0120 (18)	0.0038 (17)
C23	0.029 (2)	0.041 (3)	0.043 (2)	−0.003 (2)	0.0160 (18)	0.005 (2)
C24	0.036 (2)	0.034 (3)	0.056 (3)	0.006 (2)	0.029 (2)	0.011 (2)
C25	0.040 (3)	0.037 (3)	0.048 (3)	−0.002 (2)	0.025 (2)	−0.007 (2)
C26	0.022 (2)	0.024 (2)	0.0205 (18)	−0.0008 (18)	0.0086 (16)	−0.0017 (16)
C27	0.030 (2)	0.025 (2)	0.025 (2)	0.0003 (18)	0.0103 (17)	−0.0021 (16)
C28	0.0208 (19)	0.022 (2)	0.0223 (18)	0.0013 (17)	0.0062 (15)	0.0027 (16)
C29	0.037 (2)	0.027 (3)	0.033 (2)	−0.006 (2)	0.0155 (18)	−0.0059 (19)
C30	0.041 (3)	0.031 (3)	0.041 (2)	−0.007 (2)	0.014 (2)	0.002 (2)
C31	0.033 (2)	0.043 (3)	0.030 (2)	−0.007 (2)	0.0113 (18)	0.003 (2)
C32	0.037 (2)	0.043 (3)	0.028 (2)	−0.007 (2)	0.0195 (18)	−0.0001 (19)

### (I) N2,N3,N5,N6-Tetrakis(pyridin-2-ylmethyl)pyrazine-2,3,5,6-tetracarboxamide Geometric parameters (Å, º)

**Table:**

O1—C5	1.256 (4)	C8—H8	0.9500
O2—C12	1.220 (4)	C9—C10	1.375 (7)
O3—C19	1.246 (4)	C9—H9	0.9500
O4—C26	1.231 (4)	C10—C11	1.368 (7)
N1—C1	1.338 (4)	C10—H10	0.9500
N1—C4	1.342 (4)	C11—H11	0.9500
N2—C2	1.336 (4)	C13—C14	1.513 (5)
N2—C3	1.345 (4)	C13—H13A	0.9900
N3—C5	1.327 (4)	C13—H13B	0.9900
N3—C6	1.475 (5)	C14—C15	1.382 (5)
N3—H3N	0.97 (5)	C15—C16	1.374 (5)
N4—C7	1.338 (5)	C15—H15	0.9500
N4—C11	1.339 (6)	C16—C17	1.372 (5)
N5—C12	1.346 (5)	C16—H16	0.9500
N5—C13	1.448 (4)	C17—C18	1.378 (5)
N5—H5N	0.79 (4)	C17—H17	0.9500
N6—C18	1.339 (5)	C18—H18	0.9500
N6—C14	1.350 (4)	C20—C21	1.498 (5)
N7—C19	1.342 (4)	C20—H20A	0.9900
N7—C20	1.470 (5)	C20—H20B	0.9900
N7—H7N	0.86 (4)	C21—C22	1.385 (5)
N8—C25	1.333 (5)	C22—C23	1.374 (6)
N8—C21	1.356 (5)	C22—H22	0.9500
N9—C26	1.336 (4)	C23—C24	1.372 (6)
N9—C27	1.445 (4)	C23—H23	0.9500
N9—H9N	0.92 (4)	C24—C25	1.380 (5)
N10—C32	1.336 (5)	C24—H24	0.9500
N10—C28	1.345 (5)	C25—H25	0.9500
C1—C2	1.399 (5)	C27—C28	1.509 (5)
C1—C5	1.501 (5)	C27—H27A	0.9900
C2—C12	1.504 (5)	C27—H27B	0.9900
C3—C4	1.402 (5)	C28—C29	1.391 (5)
C3—C19	1.494 (5)	C29—C30	1.390 (5)
C4—C26	1.516 (5)	C29—H29	0.9500
C6—C7	1.511 (5)	C30—C31	1.374 (6)
C6—H6A	0.9900	C30—H30	0.9500
C6—H6B	0.9900	C31—C32	1.372 (6)
C7—C8	1.379 (6)	C31—H31	0.9500
C8—C9	1.369 (6)	C32—H32	0.9500
			
C1—N1—C4	117.5 (3)	N6—C14—C15	121.1 (4)
C2—N2—C3	118.0 (3)	N6—C14—C13	116.1 (3)
C5—N3—C6	122.0 (3)	C15—C14—C13	122.8 (3)
C5—N3—H3N	117 (3)	C16—C15—C14	119.3 (4)
C6—N3—H3N	120 (3)	C16—C15—H15	120.4
C7—N4—C11	117.5 (4)	C14—C15—H15	120.4
C12—N5—C13	121.8 (3)	C17—C16—C15	119.9 (4)
C12—N5—H5N	124 (3)	C17—C16—H16	120.0
C13—N5—H5N	114 (3)	C15—C16—H16	120.0
C18—N6—C14	118.6 (3)	C16—C17—C18	118.0 (4)
C19—N7—C20	122.9 (3)	C16—C17—H17	121.0
C19—N7—H7N	119 (3)	C18—C17—H17	121.0
C20—N7—H7N	118 (3)	N6—C18—C17	123.0 (4)
C25—N8—C21	117.0 (3)	N6—C18—H18	118.5
C26—N9—C27	122.3 (3)	C17—C18—H18	118.5
C26—N9—H9N	117 (3)	O3—C19—N7	124.7 (3)
C27—N9—H9N	118 (3)	O3—C19—C3	120.3 (3)
C32—N10—C28	117.5 (4)	N7—C19—C3	114.9 (3)
N1—C1—C2	121.6 (3)	N7—C20—C21	109.7 (3)
N1—C1—C5	114.7 (3)	N7—C20—H20A	109.7
C2—C1—C5	123.5 (3)	C21—C20—H20A	109.7
N2—C2—C1	120.7 (3)	N7—C20—H20B	109.7
N2—C2—C12	116.8 (3)	C21—C20—H20B	109.7
C1—C2—C12	122.4 (3)	H20A—C20—H20B	108.2
N2—C3—C4	120.8 (3)	N8—C21—C22	122.0 (4)
N2—C3—C19	117.0 (3)	N8—C21—C20	116.1 (3)
C4—C3—C19	122.2 (3)	C22—C21—C20	121.7 (3)
N1—C4—C3	121.1 (3)	C23—C22—C21	119.5 (4)
N1—C4—C26	113.5 (3)	C23—C22—H22	120.3
C3—C4—C26	125.3 (3)	C21—C22—H22	120.3
O1—C5—N3	125.0 (3)	C24—C23—C22	119.0 (4)
O1—C5—C1	118.9 (3)	C24—C23—H23	120.5
N3—C5—C1	115.8 (3)	C22—C23—H23	120.5
N3—C6—C7	111.8 (3)	C23—C24—C25	118.5 (4)
N3—C6—H6A	109.3	C23—C24—H24	120.7
C7—C6—H6A	109.3	C25—C24—H24	120.7
N3—C6—H6B	109.3	N8—C25—C24	123.9 (4)
C7—C6—H6B	109.3	N8—C25—H25	118.0
H6A—C6—H6B	107.9	C24—C25—H25	118.0
N4—C7—C8	122.4 (4)	O4—C26—N9	124.8 (3)
N4—C7—C6	115.8 (4)	O4—C26—C4	122.0 (3)
C8—C7—C6	121.8 (3)	N9—C26—C4	113.0 (3)
C9—C8—C7	119.2 (4)	N9—C27—C28	116.3 (3)
C9—C8—H8	120.4	N9—C27—H27A	108.2
C7—C8—H8	120.4	C28—C27—H27A	108.2
C8—C9—C10	118.9 (5)	N9—C27—H27B	108.2
C8—C9—H9	120.5	C28—C27—H27B	108.2
C10—C9—H9	120.5	H27A—C27—H27B	107.4
C11—C10—C9	118.8 (5)	N10—C28—C29	122.3 (3)
C11—C10—H10	120.6	N10—C28—C27	114.5 (3)
C9—C10—H10	120.6	C29—C28—C27	123.2 (3)
N4—C11—C10	123.2 (5)	C30—C29—C28	118.4 (4)
N4—C11—H11	118.4	C30—C29—H29	120.8
C10—C11—H11	118.4	C28—C29—H29	120.8
O2—C12—N5	125.1 (3)	C31—C30—C29	119.6 (4)
O2—C12—C2	121.5 (3)	C31—C30—H30	120.2
N5—C12—C2	113.4 (3)	C29—C30—H30	120.2
N5—C13—C14	113.0 (3)	C32—C31—C30	118.0 (4)
N5—C13—H13A	109.0	C32—C31—H31	121.0
C14—C13—H13A	109.0	C30—C31—H31	121.0
N5—C13—H13B	109.0	N10—C32—C31	124.2 (4)
C14—C13—H13B	109.0	N10—C32—H32	117.9
H13A—C13—H13B	107.8	C31—C32—H32	117.9
			
C4—N1—C1—C2	2.1 (5)	N5—C13—C14—N6	−145.5 (3)
C4—N1—C1—C5	−173.8 (3)	N5—C13—C14—C15	35.5 (5)
C3—N2—C2—C1	4.9 (5)	N6—C14—C15—C16	−2.0 (6)
C3—N2—C2—C12	−172.1 (3)	C13—C14—C15—C16	176.9 (4)
N1—C1—C2—N2	−5.0 (5)	C14—C15—C16—C17	2.8 (7)
C5—C1—C2—N2	170.5 (3)	C15—C16—C17—C18	−1.9 (6)
N1—C1—C2—C12	171.9 (3)	C14—N6—C18—C17	0.7 (6)
C5—C1—C2—C12	−12.7 (5)	C16—C17—C18—N6	0.1 (6)
C2—N2—C3—C4	−2.3 (5)	C20—N7—C19—O3	−5.1 (5)
C2—N2—C3—C19	178.5 (3)	C20—N7—C19—C3	172.8 (3)
C1—N1—C4—C3	0.5 (5)	N2—C3—C19—O3	−160.7 (3)
C1—N1—C4—C26	177.1 (3)	C4—C3—C19—O3	20.1 (5)
N2—C3—C4—N1	−0.4 (5)	N2—C3—C19—N7	21.3 (5)
C19—C3—C4—N1	178.7 (3)	C4—C3—C19—N7	−157.9 (3)
N2—C3—C4—C26	−176.5 (3)	C19—N7—C20—C21	−92.0 (4)
C19—C3—C4—C26	2.6 (5)	C25—N8—C21—C22	0.9 (6)
C6—N3—C5—O1	−2.6 (5)	C25—N8—C21—C20	−175.6 (3)
C6—N3—C5—C1	171.2 (3)	N7—C20—C21—N8	85.3 (4)
N1—C1—C5—O1	108.5 (4)	N7—C20—C21—C22	−91.2 (4)
C2—C1—C5—O1	−67.2 (5)	N8—C21—C22—C23	0.1 (6)
N1—C1—C5—N3	−65.6 (4)	C20—C21—C22—C23	176.4 (4)
C2—C1—C5—N3	118.6 (4)	C21—C22—C23—C24	−0.7 (6)
C5—N3—C6—C7	−93.0 (4)	C22—C23—C24—C25	0.3 (6)
C11—N4—C7—C8	−1.9 (6)	C21—N8—C25—C24	−1.4 (6)
C11—N4—C7—C6	180.0 (4)	C23—C24—C25—N8	0.8 (7)
N3—C6—C7—N4	−101.2 (4)	C27—N9—C26—O4	−5.2 (6)
N3—C6—C7—C8	80.6 (5)	C27—N9—C26—C4	168.9 (3)
N4—C7—C8—C9	1.5 (7)	N1—C4—C26—O4	81.6 (4)
C6—C7—C8—C9	179.5 (4)	C3—C4—C26—O4	−102.1 (4)
C7—C8—C9—C10	−0.7 (8)	N1—C4—C26—N9	−92.6 (4)
C8—C9—C10—C11	0.4 (8)	C3—C4—C26—N9	83.7 (4)
C7—N4—C11—C10	1.6 (7)	C26—N9—C27—C28	97.9 (4)
C9—C10—C11—N4	−0.9 (8)	C32—N10—C28—C29	−0.9 (5)
C13—N5—C12—O2	−2.3 (5)	C32—N10—C28—C27	178.6 (3)
C13—N5—C12—C2	175.4 (3)	N9—C27—C28—N10	174.1 (3)
N2—C2—C12—O2	166.2 (3)	N9—C27—C28—C29	−6.4 (5)
C1—C2—C12—O2	−10.7 (5)	N10—C28—C29—C30	−0.4 (5)
N2—C2—C12—N5	−11.6 (4)	C27—C28—C29—C30	−179.8 (3)
C1—C2—C12—N5	171.5 (3)	C28—C29—C30—C31	1.4 (6)
C12—N5—C13—C14	−97.1 (4)	C29—C30—C31—C32	−1.1 (6)
C18—N6—C14—C15	0.3 (5)	C28—N10—C32—C31	1.2 (6)
C18—N6—C14—C13	−178.7 (3)	C30—C31—C32—N10	−0.2 (6)

### (I) N2,N3,N5,N6-Tetrakis(pyridin-2-ylmethyl)pyrazine-2,3,5,6-tetracarboxamide Hydrogen-bond geometry (Å, º)

**Table:**

*D*—H···*A*	*D*—H	H···*A*	*D*···*A*	*D*—H···*A*
C29—H29···O3	0.95	2.48	3.389 (5)	160
N3—H3*N*···O3^i^	0.97 (5)	1.91 (5)	2.829 (4)	158 (4)
N5—H5*N*···O1^ii^	0.79 (4)	2.17 (4)	2.932 (4)	162 (3)
N7—H7*N*···O1^ii^	0.86 (4)	2.14 (4)	2.967 (4)	161 (4)
N9—H9*N*···N6^iii^	0.92 (4)	1.96 (5)	2.864 (4)	169 (5)
C13—H13*A*···N1^ii^	0.99	2.62	3.554 (5)	158
C20—H20*A*···O2^ii^	0.99	2.54	3.433 (4)	149
C22—H22···O2^ii^	0.95	2.57	3.418 (5)	149

Symmetry codes: (i) −*x*, −*y*+1, −*z*; (ii) −*x*+1/2, *y*+1/2, −*z*+1/2; (iii) −*x*+1/2, *y*−1/2, −*z*+1/2.

### (II) N2,N3,N5,N6-Tetrakis(pyridin-4-ylmethyl)pyrazine-2,3,5,6-tetracarboxamide Crystal data

**Table:**

C_32_H_28_N_10_O_4_	*F*(000) = 644
*M**_r_* = 616.64	*D*_x_ = 1.348 Mg m^−^^3^
Monoclinic, *P*2_1_/*n*	Mo *K*α radiation, λ = 0.71073 Å
*a* = 9.8592 (6) Å	Cell parameters from 7048 reflections
*b* = 10.6511 (6) Å	θ = 2.3–25.9°
*c* = 14.8089 (9) Å	µ = 0.09 mm^−^^1^
β = 102.306 (7)°	*T* = 153 K
*V* = 1519.37 (16) Å^3^	Block, colourless
*Z* = 2	0.45 × 0.35 × 0.20 mm

### (II) N2,N3,N5,N6-Tetrakis(pyridin-4-ylmethyl)pyrazine-2,3,5,6-tetracarboxamide Data collection

**Table:**

Stoe IPDS 1 diffractometer	2924 independent reflections
Radiation source: fine-focus sealed tube	1815 reflections with *I* > 2σ(*I*)
Plane graphite monochromator	*R*_int_ = 0.090
φ rotation scans	θ_max_ = 25.9°, θ_min_ = 2.3°
Absorption correction: multi-scan (MULABS in PLATON; Spek, 2009)	*h* = −12→12
*T*_min_ = 0.666, *T*_max_ = 1.000	*k* = −13→13
11450 measured reflections	*l* = −18→18

### (II) N2,N3,N5,N6-Tetrakis(pyridin-4-ylmethyl)pyrazine-2,3,5,6-tetracarboxamide Refinement

**Table:**

Refinement on *F*^2^	Primary atom site location: structure-invariant direct methods
Least-squares matrix: full	Secondary atom site location: difference Fourier map
*R*[*F*^2^ > 2σ(*F*^2^)] = 0.051	Hydrogen site location: mixed
*wR*(*F*^2^) = 0.134	H atoms treated by a mixture of independent and constrained refinement
*S* = 0.89	*w* = 1/[σ^2^(*F*_o_^2^) + (0.0843*P*)^2^] where *P* = (*F*_o_^2^ + 2*F*_c_^2^)/3
2924 reflections	(Δ/σ)_max_ = 0.001
244 parameters	Δρ_max_ = 0.26 e Å^−^^3^
0 restraints	Δρ_min_ = −0.24 e Å^−^^3^

### (II) N2,N3,N5,N6-Tetrakis(pyridin-4-ylmethyl)pyrazine-2,3,5,6-tetracarboxamide Special details

**Table:**

Geometry. All esds (except the esd in the dihedral angle between two l.s. planes) are estimated using the full covariance matrix. The cell esds are taken into account individually in the estimation of esds in distances, angles and torsion angles; correlations between esds in cell parameters are only used when they are defined by crystal symmetry. An approximate (isotropic) treatment of cell esds is used for estimating esds involving l.s. planes.

### (II) N2,N3,N5,N6-Tetrakis(pyridin-4-ylmethyl)pyrazine-2,3,5,6-tetracarboxamide Fractional atomic coordinates and isotropic or equivalent isotropic displacement parameters (Å^2^)

**Table:**

	*x*	*y*	*z*	*U*_iso_*/*U*_eq_	Occ. (<1)
N1	−0.03421 (18)	0.58335 (15)	0.92721 (11)	0.0272 (4)	
N2	0.22670 (19)	0.60099 (18)	0.84892 (13)	0.0302 (4)	
H2N	0.244 (3)	0.658 (3)	0.898 (2)	0.055 (8)*	
N4	−0.2443 (2)	0.74564 (17)	0.91134 (12)	0.0299 (4)	
H4N	−0.182 (3)	0.738 (2)	0.8753 (18)	0.040 (7)*	
N5	−0.2772 (2)	1.19617 (19)	1.02333 (13)	0.0430 (5)	
O1	0.09946 (16)	0.43306 (14)	0.78401 (10)	0.0357 (4)	
O2	−0.29807 (17)	0.67310 (16)	1.04302 (10)	0.0435 (5)	
C1	0.0706 (2)	0.50299 (19)	0.93128 (13)	0.0251 (5)	
C2	−0.1056 (2)	0.58116 (19)	0.99495 (13)	0.0257 (5)	
C3	0.1375 (2)	0.5078 (2)	0.84783 (13)	0.0264 (5)	
C4	0.2840 (2)	0.6340 (2)	0.76899 (15)	0.0350 (5)	
H4A	0.298850	0.725974	0.769395	0.042*	
H4B	0.214362	0.613342	0.712270	0.042*	
C5	0.4176 (2)	0.5708 (2)	0.76382 (14)	0.0369 (6)	
C6	0.5084 (2)	0.6284 (3)	0.71674 (16)	0.0428 (6)	
H6	0.488582	0.710018	0.691424	0.051*	
C7	0.6260 (3)	0.5674 (4)	0.7070 (2)	0.0638 (9)	
H7	0.695836	0.611527	0.684660	0.077*	
N3A	0.6459 (12)	0.4293 (17)	0.7318 (7)	0.061 (3)	0.58 (3)
C8A	0.5525 (14)	0.3778 (16)	0.7738 (7)	0.057 (3)	0.58 (3)
H8A	0.565436	0.293085	0.793745	0.068*	0.58 (3)
C9A	0.4378 (16)	0.4420 (15)	0.7897 (9)	0.049 (2)	0.58 (3)
H9A	0.372404	0.400153	0.817822	0.059*	0.58 (3)
N3B	0.6817 (19)	0.4827 (19)	0.7509 (12)	0.055 (4)	0.42 (3)
C8B	0.603 (2)	0.429 (2)	0.8028 (15)	0.061 (5)	0.42 (3)
H8B	0.638613	0.355212	0.835676	0.073*	0.42 (3)
C9B	0.474 (2)	0.469 (2)	0.8135 (13)	0.045 (4)	0.42 (3)
H9B	0.426497	0.427346	0.854273	0.054*	0.42 (3)
C10	−0.2250 (2)	0.67050 (19)	0.98583 (14)	0.0292 (5)	
C11	−0.3563 (2)	0.8358 (2)	0.89116 (14)	0.0302 (5)	
H11A	−0.439256	0.798804	0.908574	0.036*	
H11B	−0.379534	0.850972	0.823670	0.036*	
C12	−0.3255 (2)	0.9596 (2)	0.93967 (13)	0.0280 (5)	
C13	−0.4219 (2)	1.0556 (2)	0.91948 (15)	0.0335 (5)	
H13	−0.506721	1.042206	0.876122	0.040*	
C14	−0.3951 (2)	1.1701 (2)	0.96208 (16)	0.0377 (6)	
H14	−0.463442	1.234122	0.947499	0.045*	
C15	−0.1850 (3)	1.1033 (2)	1.04220 (17)	0.0462 (6)	
H15	−0.100541	1.119381	1.085262	0.055*	
C16	−0.2041 (2)	0.9855 (2)	1.00334 (15)	0.0374 (6)	
H16	−0.134841	0.922753	1.020004	0.045*	

### (II) N2,N3,N5,N6-Tetrakis(pyridin-4-ylmethyl)pyrazine-2,3,5,6-tetracarboxamide Atomic displacement parameters (Å^2^)

**Table:**

	*U*^11^	*U*^22^	*U*^33^	*U*^12^	*U*^13^	*U*^23^
N1	0.0310 (9)	0.0273 (10)	0.0236 (8)	0.0014 (8)	0.0069 (7)	−0.0008 (7)
N2	0.0327 (10)	0.0303 (11)	0.0297 (9)	−0.0008 (8)	0.0114 (8)	−0.0023 (8)
N4	0.0361 (10)	0.0311 (11)	0.0236 (9)	0.0061 (8)	0.0087 (8)	0.0028 (7)
N5	0.0529 (13)	0.0374 (12)	0.0396 (11)	0.0010 (10)	0.0121 (10)	−0.0045 (9)
O1	0.0419 (9)	0.0411 (9)	0.0250 (8)	−0.0056 (7)	0.0090 (6)	−0.0069 (7)
O2	0.0489 (10)	0.0523 (11)	0.0356 (9)	0.0206 (8)	0.0228 (8)	0.0139 (7)
C1	0.0293 (11)	0.0238 (11)	0.0224 (10)	−0.0008 (9)	0.0060 (8)	−0.0023 (8)
C2	0.0294 (11)	0.0252 (11)	0.0230 (10)	0.0006 (9)	0.0063 (8)	−0.0019 (8)
C3	0.0269 (11)	0.0284 (12)	0.0244 (10)	0.0051 (9)	0.0063 (8)	0.0011 (8)
C4	0.0346 (12)	0.0404 (13)	0.0321 (11)	−0.0008 (11)	0.0113 (9)	0.0066 (10)
C5	0.0378 (13)	0.0516 (16)	0.0224 (11)	0.0022 (11)	0.0088 (9)	−0.0042 (10)
C6	0.0399 (13)	0.0584 (16)	0.0330 (12)	−0.0071 (12)	0.0138 (10)	−0.0140 (11)
C7	0.0480 (17)	0.107 (3)	0.0429 (16)	0.0061 (18)	0.0232 (13)	−0.0043 (17)
N3A	0.053 (4)	0.085 (7)	0.052 (4)	0.023 (4)	0.027 (3)	0.000 (4)
C8A	0.063 (5)	0.074 (6)	0.039 (4)	0.026 (5)	0.024 (4)	0.011 (4)
C9A	0.045 (6)	0.072 (6)	0.031 (5)	0.017 (4)	0.011 (4)	0.005 (4)
N3B	0.062 (7)	0.062 (8)	0.052 (6)	0.019 (6)	0.035 (5)	0.013 (5)
C8B	0.066 (8)	0.070 (9)	0.055 (8)	0.032 (7)	0.029 (7)	0.027 (7)
C9B	0.048 (8)	0.068 (9)	0.025 (6)	0.023 (6)	0.019 (5)	0.022 (6)
C10	0.0352 (12)	0.0274 (12)	0.0258 (10)	0.0036 (9)	0.0082 (9)	0.0012 (8)
C11	0.0312 (11)	0.0311 (12)	0.0266 (10)	0.0047 (9)	0.0023 (8)	0.0034 (9)
C12	0.0313 (12)	0.0313 (12)	0.0223 (10)	0.0032 (9)	0.0074 (8)	0.0037 (8)
C13	0.0308 (12)	0.0345 (13)	0.0347 (12)	0.0047 (10)	0.0059 (9)	0.0042 (10)
C14	0.0401 (13)	0.0349 (13)	0.0413 (12)	0.0090 (11)	0.0158 (10)	0.0047 (10)
C15	0.0470 (15)	0.0456 (16)	0.0408 (14)	0.0020 (12)	−0.0021 (11)	−0.0085 (11)
C16	0.0375 (13)	0.0367 (14)	0.0337 (12)	0.0078 (11)	−0.0018 (10)	−0.0019 (10)

### (II) N2,N3,N5,N6-Tetrakis(pyridin-4-ylmethyl)pyrazine-2,3,5,6-tetracarboxamide Geometric parameters (Å, º)

**Table:**

N1—C1	1.333 (3)	C7—N3B	1.177 (12)
N1—C2	1.343 (3)	C7—N3A	1.518 (17)
N2—C3	1.324 (3)	C7—H7	0.9500
N2—C4	1.459 (3)	N3A—C8A	1.334 (12)
N2—H2N	0.93 (3)	C8A—C9A	1.384 (16)
N4—C10	1.343 (3)	C8A—H8A	0.9500
N4—C11	1.446 (3)	C9A—H9A	0.9500
N4—H4N	0.90 (3)	N3B—C8B	1.336 (16)
N5—C15	1.333 (3)	C8B—C9B	1.38 (2)
N5—C14	1.342 (3)	C8B—H8B	0.9500
O1—C3	1.232 (2)	C9B—H9B	0.9500
O2—C10	1.224 (3)	C11—C12	1.502 (3)
C1—C2^i^	1.398 (3)	C11—H11A	0.9900
C1—C3	1.521 (3)	C11—H11B	0.9900
C2—C10	1.497 (3)	C12—C16	1.384 (3)
C4—C5	1.495 (3)	C12—C13	1.385 (3)
C4—H4A	0.9900	C13—C14	1.373 (3)
C4—H4B	0.9900	C13—H13	0.9500
C5—C9B	1.360 (18)	C14—H14	0.9500
C5—C6	1.389 (3)	C15—C16	1.376 (3)
C5—C9A	1.427 (16)	C15—H15	0.9500
C6—C7	1.363 (4)	C16—H16	0.9500
C6—H6	0.9500		
			
C1—N1—C2	118.66 (17)	N3A—C8A—H8A	118.4
C3—N2—C4	122.83 (19)	C9A—C8A—H8A	118.4
C3—N2—H2N	120.4 (17)	C8A—C9A—C5	120.1 (12)
C4—N2—H2N	115.9 (17)	C8A—C9A—H9A	120.0
C10—N4—C11	122.25 (19)	C5—C9A—H9A	120.0
C10—N4—H4N	116.1 (16)	C7—N3B—C8B	112.7 (11)
C11—N4—H4N	121.6 (16)	N3B—C8B—C9B	126.5 (12)
C15—N5—C14	116.3 (2)	N3B—C8B—H8B	116.7
N1—C1—C2^i^	120.44 (19)	C9B—C8B—H8B	116.7
N1—C1—C3	114.06 (17)	C5—C9B—C8B	117.8 (13)
C2^i^—C1—C3	125.39 (18)	C5—C9B—H9B	121.1
N1—C2—C1^i^	120.89 (19)	C8B—C9B—H9B	121.1
N1—C2—C10	116.66 (17)	O2—C10—N4	123.6 (2)
C1^i^—C2—C10	122.44 (18)	O2—C10—C2	121.30 (18)
O1—C3—N2	125.93 (19)	N4—C10—C2	115.08 (19)
O1—C3—C1	119.20 (19)	N4—C11—C12	114.61 (17)
N2—C3—C1	114.69 (18)	N4—C11—H11A	108.6
N2—C4—C5	115.50 (18)	C12—C11—H11A	108.6
N2—C4—H4A	108.4	N4—C11—H11B	108.6
C5—C4—H4A	108.4	C12—C11—H11B	108.6
N2—C4—H4B	108.4	H11A—C11—H11B	107.6
C5—C4—H4B	108.4	C16—C12—C13	117.0 (2)
H4A—C4—H4B	107.5	C16—C12—C11	123.95 (19)
C9B—C5—C6	113.0 (8)	C13—C12—C11	119.04 (18)
C6—C5—C9A	119.4 (6)	C14—C13—C12	120.0 (2)
C9B—C5—C4	126.4 (8)	C14—C13—H13	120.0
C6—C5—C4	119.7 (2)	C12—C13—H13	120.0
C9A—C5—C4	119.6 (6)	N5—C14—C13	123.2 (2)
C7—C6—C5	119.7 (3)	N5—C14—H14	118.4
C7—C6—H6	120.1	C13—C14—H14	118.4
C5—C6—H6	120.1	N5—C15—C16	124.2 (2)
N3B—C7—C6	128.0 (6)	N5—C15—H15	117.9
C6—C7—N3A	120.4 (5)	C16—C15—H15	117.9
C6—C7—H7	119.8	C15—C16—C12	119.2 (2)
N3A—C7—H7	119.8	C15—C16—H16	120.4
C8A—N3A—C7	116.4 (7)	C12—C16—H16	120.4
N3A—C8A—C9A	123.1 (10)		
			
C2—N1—C1—C2^i^	−0.4 (3)	C4—C5—C9A—C8A	171.5 (5)
C2—N1—C1—C3	176.06 (18)	C6—C7—N3B—C8B	13.7 (16)
C1—N1—C2—C1^i^	0.4 (3)	C7—N3B—C8B—C9B	−5.4 (18)
C1—N1—C2—C10	−178.23 (17)	C6—C5—C9B—C8B	−7.3 (13)
C4—N2—C3—O1	5.4 (3)	C4—C5—C9B—C8B	−176.3 (10)
C4—N2—C3—C1	−169.63 (18)	N3B—C8B—C9B—C5	3.0 (19)
N1—C1—C3—O1	−95.4 (2)	C11—N4—C10—O2	−1.2 (3)
C2^i^—C1—C3—O1	80.8 (3)	C11—N4—C10—C2	178.84 (18)
N1—C1—C3—N2	80.0 (2)	N1—C2—C10—O2	178.2 (2)
C2^i^—C1—C3—N2	−103.8 (2)	C1^i^—C2—C10—O2	−0.4 (3)
C3—N2—C4—C5	−91.4 (2)	N1—C2—C10—N4	−1.8 (3)
N2—C4—C5—C9B	14.7 (13)	C1^i^—C2—C10—N4	179.59 (19)
N2—C4—C5—C6	−153.7 (2)	C10—N4—C11—C12	84.7 (3)
N2—C4—C5—C9A	39.5 (7)	N4—C11—C12—C16	−3.9 (3)
C9B—C5—C6—C7	14.2 (11)	N4—C11—C12—C13	175.06 (19)
C9A—C5—C6—C7	−9.1 (7)	C16—C12—C13—C14	−0.1 (3)
C4—C5—C6—C7	−175.9 (2)	C11—C12—C13—C14	−179.1 (2)
C5—C6—C7—N3B	−19.6 (17)	C15—N5—C14—C13	−0.5 (4)
C5—C6—C7—N3A	11.0 (7)	C12—C13—C14—N5	0.6 (4)
C6—C7—N3A—C8A	−8.4 (9)	C14—N5—C15—C16	−0.1 (4)
C7—N3A—C8A—C9A	4.0 (11)	N5—C15—C16—C12	0.6 (4)
N3A—C8A—C9A—C5	−2.3 (11)	C13—C12—C16—C15	−0.5 (3)
C6—C5—C9A—C8A	4.6 (9)	C11—C12—C16—C15	178.5 (2)

Symmetry code: (i) −*x*, −*y*+1, −*z*+2.

### (II) N2,N3,N5,N6-Tetrakis(pyridin-4-ylmethyl)pyrazine-2,3,5,6-tetracarboxamide Hydrogen-bond geometry (Å, º)

**Table:**

*D*—H···*A*	*D*—H	H···*A*	*D*···*A*	*D*—H···*A*
C9*A*—H9*A*···O2^i^	0.95	2.46	3.316 (15)	150
[C9*B*—H9*B*···O2]^i^	0.95	2.43	3.375 (18)	178
N2—H2*N*···N5^ii^	0.93 (3)	1.93 (3)	2.845 (3)	167 (2)
N4—H4*N*···N3*A*^iii^	0.90 (3)	2.65 (3)	3.184 (13)	119 (2)
C6—H6···O1^iii^	0.95	2.58	3.414 (3)	146
C11—H11*B*···O1^iv^	0.99	2.56	3.301 (2)	132
C14—H14···O2^v^	0.95	2.58	3.442 (3)	151

Symmetry codes: (i) −*x*, −*y*+1, −*z*+2; (ii) −*x*, −*y*+2, −*z*+2; (iii) −*x*+1/2, *y*+1/2, −*z*+3/2; (iv) −*x*−1/2, *y*+1/2, −*z*+3/2; (v) −*x*−1, −*y*+2, −*z*+2.
